# Egg-driven immunosuppression and granuloma zonation in Peyer’s patches of mice with *Schistosoma japonicum* infection

**DOI:** 10.3389/fcimb.2025.1587166

**Published:** 2025-04-29

**Authors:** Linzhu Li, Jing Wu, Guangxu Cao, Jiakai Yao, Yanping Miao, Yanglin Zhuang, Yushen Xiang, Xiaolin Zhong, Yicong Liu, Fubo Chen, Yalei Dai, Yang Dai, Xindong Xu, Qingfeng Zhang

**Affiliations:** ^1^ Laboratory of Molecular Parasitology, State Key Laboratory of Cardiology and Research Center for Translational Medicine, Shanghai East Hospital, Key Laboratory of Pathogen-Host Interaction (Tongji University), Ministry of Education, Clinical Center for Brain and Spinal Cord Research, School of Medicine, Tongji University, Shanghai, China; ^2^ Department of Gynecology, School of Medicine, Shanghai East Hospital, Tongji University, Shanghai, China; ^3^ National Health Commission Key Laboratory of Parasitic Disease Control and Prevention, Jiangsu Provincial Key Laboratory on Parasite and Vector Control Technology, Jiangsu Institute of Parasitic Diseases, Wuxi, China; ^4^ The Fifth People’s Hospital of Jiangxi Province, Nanchang, Jiangxi, China; ^5^ Jiangxi Provincial Blood Center, Nanchang, Jiangxi, China; ^6^ Department of Medical Ultrasound and Department of Stomatology, Shanghai Tenth People’s Hospital, Tongji University School of Medicine, Tongji University, Shanghai, China; ^7^ Shanghai Key Lab of Tuberculosis, Shanghai Pulmonary Hospital, and Department of Immunology and Microbiology, Tongji University School of Medicine, Shanghai, China

**Keywords:** *Schistosoma japonicum*, granuloma, Peyer’s patch, immunosuppression, fibrosis

## Abstract

Egg granulomas caused by *Schistosoma japonicum* (*S. japonicum*) are important causes of morbidity and mortality in schistosomiasis. The intestine plays a crucial role in the complete life cycle of *S. japonicum*; eggs are transported through the intestine and excreted with feces. During this process, the interaction between the eggs and the intestine can trigger a strong intestinal immune system response and cause inflammation. Eggs in the intestine preferentially accumulate in Peyer’s patches (PPs). However, the cellular composition of intestinal granulomas and the impacts of egg deposition on the immune function of PPs remain poorly understood. Using a mouse model of *S. japonicum* infection, we revealed that the deposition of eggs disrupted the structure of PPs, resulting in immunosuppression. We further characterized the cellular composition of intestinal granulomas, revealing a layered distribution of neutrophils, macrophages, T cells, and B cells, with marked neutrophil accumulation. Single-cell RNA sequencing revealed that egg deposition drives B-cell apoptosis, T-cell exhaustion, and activation of fibrotic pathways in myeloid cells, collectively impairing PP function. In conclusion, the layered cellular architecture of intestinal granulomas in PPs suggests a unique immune microenvironment of egg-driven immunosuppression and fibrotic remodeling, and the identification of fibrotic pathways in myeloid cells provides a potential therapeutic target to alleviate fibrosis in patients with *S. japonicum* infection.

## 1 Introduction

Human schistosomiasis is a parasitic disease caused by the genus *Schistosoma*, with an estimated global infection rate exceeding 200 million people (Cornes, 1965; Colley et al., 2014; Barnett, 2018). Intestinal schistosomiasis is caused mainly by *Schistosoma japonicum* (*S. japonicum*) and *Schistosoma mansoni* (*S. mansoni*). Once the schistosomes mature, male and female worms embrace and lay eggs in the mesenteric veins, with a theoretical reproductive potential of up to 600 billion eggs per pair of Schistosoma. *S. mansoni* lays approximately 200–300 eggs daily, while female *S. japonicum* can produce up to 3,500 eggs, with most eggs deposited in the liver and intestine (Ross et al., 2001; Turner et al., 2012).

Egg-induced granulomas are a primary factor in *S. japonicum* pathogenesis. Eggs secrete soluble egg antigens (SEAs), which stimulate resident macrophages to secrete inflammatory cytokines and chemokines and recruit neutrophils, monocytes, B cells, and T cells, thereby triggering granulomatous inflammation (McManus et al., 2018; Zheng et al., 2020). During infection, *S. japonicum* eggs recruit numerous neutrophils to encapsulate the eggs, with these neutrophils at the granuloma core releasing proinflammatory cytokines, leading to tissue damage (Von Lichtenberg et al., 1973; Chuah et al., 2013). In the chronic infection stage, there is a significant increase in B cells in the lymph nodes and spleen, which secrete a mixture of IgG1, IgG2, IgG3, and IgA targeted at egg-induced granulomas, promoting a Th2-type response (El-Cheikhi et al., 1998; Harris and Gause, 2011; Zheng et al., 2020). In addition, SEA induces helper T-cell 2 (Th2) cells to exert anti-inflammatory effects by secreting the Th2 cytokines IL-4, IL-5, and IL-13 (Pearce et al., 1991). These cytokines polarize macrophages into alternatively activated macrophages (M2), inducing the expression of arginase-1 (Arg-1), Ym-1, and Fizz1, thereby exerting immunosuppressive effects and promoting egg-induced fibrosis (Gordon and Martinez, 2010).

Peyer’s patches (PPs) are vital components of gut-associated lymphoid tissue and serve as critical initiation sites for adaptive immune responses in the intestine. In humans, the number and size of PPs vary with age, averaging approximately 240 during puberty and approximately 100–200 in other age groups (Cornes, 1965). PPs are key defense sites against microbial pathogens in the intestine and are the preferred deposition site for *Schistosoma* eggs (Cerutti and Rescigno, 2008; Turner et al., 2012). Studies have shown that after *Schistosoma* infection, the area of PPs and the size of B-cell follicles progressively decrease with the deposition of eggs, indicating that egg deposition has an adverse effect on PP homeostasis (Turner et al., 2012). However, the loss of gut-associated lymphoid tissue (GALT) integrity is limited to PPs, and mesenteric lymph nodes (mLNs) retain intact microstructures (Turner et al., 2012). However, the cellular composition of intestinal granulomas and the impacts of egg deposition on the immune function of PPs remain poorly understood.

Despite extensive characterization of *S. japonicum*-induced hepatic pathology, the immunopathogenic impact of egg deposition on gut-associated lymphoid tissues remains poorly defined. In this study, we characterized the cellular composition and functional changes in PPs following *S. japonicum* egg deposition via single-cell RNA sequencing and multiplex immunofluorescence. The research revealed that the fibrosis process is driven primarily by myeloid cells that are not present in PPs. This study is the first to elucidate the composition of intestinal granulomas induced by *S. japonicum* and identify detrimental effects on immune cells, including B-cell apoptosis and T-cell exhaustion.

## 2 Methods

### 2.1 Schistosomiasis japonicum mouse model and ethics

Female C57BL/6 mice (6 weeks old, 20–25 g) were purchased from Beijing Siberian Biotechnology Co., Ltd. Mice were housed under specific pathogen-free (SPF) conditions in an ABSL-2 facility at Tongji University with *ad libitum* access to autoclaved food and water. Animals were randomly assigned to two groups (n = 5/group): the normal healthy control (HC) group and the *S. japonicum*-infected (Infected) group. In the infected group, the mice were percutaneously infected with 30 ± 2 cercariae (*S. japonicum* Guichi strain) via exposure of the abdominal skin to cercariae-coated coverslips. At 42 days post-infection (dpi), the mice were euthanized via CO_2_ asphyxiation followed by cervical dislocation. Systemic perfusion with pH 7.4 phosphate-buffered saline (PBS) (10010072, Gibco, the same product was used throughout the experiment) was performed prior to the collection of intestinal Peyer’s patches and the liver for downstream analyses. All experimental procedures were approved by the Institutional Animal Care and Use Committee (IACUC) of Tongji University (Approval No. TJAA11422101).

### 2.2 Isolation and collection of Peyer’s patches

After euthanizing mice, abdominal cavity was opened through a midline incision, and the entire small intestine was carefully excised and placed in ice-cold PBS. The intestine was flushed with 20 ml of cold PBS to remove the luminal contents. Using a stereomicroscope, Peyer’s patches were identified as oval, dome-shaped lymphoid structures along the antimesenteric border of the small intestine. Fine-tipped forceps and micro-scissors were used to dissect PPs. The PPs were immediately transferred to RPMI 1640 medium supplemented with 10% fetal bovine serum (FBS) for further processing or analysis.

### 2.3 Hematoxylin and eosin staining

Peyer’s patches were fixed in 4% paraformaldehyde (PFA) for 24–48 h at 4°C, dehydrated through a graded ethanol series (70%, 80%, 95%, and 100%), and embedded in paraffin. Sections were cut at a thickness of 4–5 μm using a microtome (Leica RM2235) and mounted on poly-L-lysine-coated slides. For staining, the sections were deparaffinized in xylene and rehydrated through a descending ethanol series (100%, 95%, 80%, and 70%). Nuclei were stained with Harris hematoxylin for 5–8 min, followed by rinsing in running tap water for 5 min. Cytoplasmic staining was performed with 0.5% eosin Y for 1–2 min. After dehydration through an ascending ethanol series and clearing in xylene, the slides were mounted with neutral balsam and imaged under a light microscope (Nikon Eclipse E100).

### 2.4 Masson staining

Paraffin sections (4-μm thick) were deparaffinized using xylene substitutes (Eco-friendly dewaxing solution I/II, Servicebio). The deparaffinization process involved two 20-min incubations. Subsequently, the sections were stepwise rehydrated in graded ethanol solutions (100% ethanol for two changes, 95%, 80%, and 70%), with each gradient treatment lasting 5 minutes. After 5 min of washing in running tap water, the sections were subjected to nuclear staining with Weigert’s iron hematoxylin (Component A of the G1006 kit) for 10 min at 25°C. Following differentiation in 1% acid ethanol and bluing in Scott’s tap water substitute, we sequentially stained the section with Biebrich scarlet-acid fuchsin (5 min) and aniline blue (8 min) according to Masson’s Trichrome Staining Kit protocol (G1006, Servicebio). Critical differentiation steps were performed in 1% phosphomolybdic acid (2 min) and 1% acetic acid (1 min). Finally, the sections were dehydrated through graded ethanol (95%, 100% ×2), cleared in xylene (3 × 5 min), and mounted with neutral balsam. Collagen quantification was performed via ImageJ v1.53t with threshold-based area fraction analysis (blue channel, 480–500 nm).

### 2.5 Immunohistochemistry

Paraffin-embedded sections (4 μm) were deparaffinized with xylene substitutes (Eco-friendly solutions I–III, 10 min each) and rehydrated through a graded ethanol series (100% ×3, 95%, 80%, 5 min/step). Endogenous peroxidase activity was quenched with 3% H_2_O_2_ in methanol (25 min, room temperature, dark). After being washed three times with PBS (pH 7.4, 5 min per wash), the samples were subjected to heat-induced epitope retrieval in 10 mM citrate buffer (pH 6.0, 95°C, 20 min). Subsequently, it was blocked with 3% bovine serum albumin (BSA) (GC305010, Servicebio) for 30 minutes at 25°C. The primary antibody incubation was carried out using a rabbit anti-collagen I polyclonal antibody (1:200, GB11022-3, Servicebio). The incubation was conducted overnight at 4°C in a humidified chamber. The sections were then incubated with HRP-conjugated goat anti-rabbit IgG (1:500, G1302, Servicebio) for 1 h at 25°C. DAB chromogen (G1212, Servicebio) was applied until signal emergence (2–5 min) under microscopic monitoring, followed by hematoxylin counterstaining (3 min). The sections were differentiated in 0.5% acid ethanol (10 s), blued in Scott’s buffer (pH 8.0, 5 min), and dehydrated through a graded ethanol/xylene mixture. The slides were mounted using Permount™ and then imaged with a Nikon Eclipse Ni-U microscope under 20× objective. Quantitative analysis was performed via ImageJ v1.53t using threshold-based positive pixel quantification (brown channel, 550–600 nm) across three randomly selected fields per section by blinded investigators.

### 2.6 Quantitative real-time PCR

Total RNA from Peyer’s patches was extracted using TRIzol reagent (Invitrogen) according to the manufacturer’s instructions. cDNA synthesis was performed with 1 μg of total RNA using HiScript^®^ III RT SuperMix (+gDNA wiper) (Vazyme, China) to remove genomic DNA contamination. Quantitative PCR (qPCR) amplification was carried out on a QuantStudio 6 Pro System (Applied Biosystems, USA) with SYBR Green Premix (Vazyme) using validated primer pairs (see **Supplementary Table S1**). All reactions were conducted in technical triplicate with the following cycling conditions: 95°C for 30 s, followed by 40 cycles of 95°C for 10 s and 60°C for 30 s. Gene expression levels were normalized to those of GAPDH and calculated using the 2^−ΔΔCT^ method (Livak and Schmittgen, 2001).

### 2.7 Transcriptome sequencing

PP total RNA with an RNA integrity number (RIN) >8.0 (Agilent Bioanalyzer 2100) was used for stranded mRNA-seq library preparation via the NEBNext Ultra II RNA Library Prep Kit (New England Biolabs). Libraries were quantified with a Qubit 4.0 fluorometer (Thermo Fisher), and the size distribution was validated using an Agilent 2100 TapeStation (D1000 ScreenTape). Cluster generation was performed on a cBot 2 System (Illumina) with a TruSeq PE Cluster Kit v3 (150 bp paired-end mode). Sequencing was carried out on an Illumina NovaSeq 6000 platform (2×150 bp) to achieve ≥20 million reads per sample.

Quality control was carried out using FastQC v0.11.9, and adapter removal as well as low-quality base trimming was performed with Trimmomatic v0.39 (SLIDINGWINDOW:4:20, MINLEN:50). Clean reads were aligned to the GRCm38 (mm10) reference genome via STAR aligner v2.7.10b with default parameters. Differential gene expression between the normal healthy control (HC) and *S. japonicum*-infected (Infected) groups was analyzed using DESeq2 v1.36.0 in R. The significance thresholds were set at a Log2 fold-change (L2FC) greater than 1 and a Benjamini–Hochberg (BH)-adjusted P-value less than 0.05. Functional enrichment analysis of differentially expressed genes (DEGs) was conducted via clusterProfiler v4.6.2, with Kyoto Encyclopedia of Genes and Genomes (KEGG) pathways considered significantly enriched at a false discovery rate (FDR) < 0.05.

### 2.8 Multiplex immunofluorescence

The procedures for section sample preparation and antigen retrieval were identical to those used in immunohistochemistry. Multiplex immunofluorescence (mIF) was performed using a five-color kit (TGFP550, Tissuegnostics) to detect CD19, CD3, F4/80, Ly6G, and DAPI, following the manufacturer’s instructions at Tissuegnostics Asia Pacific Limited (Beijing, China). Immunofluorescence images were acquired via the Tissue FAXS Cytometry platform (Tissuegnostics) and analyzed with StrataQuest software (version 7.1.6) for high-throughput quantification. A fluorescence microscope was used to detect signals in five distinct channels: blue for DAPI (nuclear stain), orange for CD19 (1:500 dilution, ab245235, Abcam, Cambridge, USA), red for CD3 (1:1500 dilution, ab237721, Abcam, Cambridge, USA), green for F4/80 (1:500 dilution, GB113373-100, Servicebio, Wuhan, China), and purple for Ly6G (1:200 dilution, GB11229-100, Servicebio, Wuhan, China). The cell nuclei were identified via the DAPI fluorescence channel, and the parameters were optimized using the “forward–reverse track tools” in Strata Quest to determine the total cell number per field of view (n/sight) via quantitative analysis. The nuclear and cytoplasmic regions were then segmented on the basis of clear fluorescence signals. Optimal positive thresholds for each marker (CD19, CD3, F4/80, and Ly6G) were established by assessing the fluorescence signal intensity around the nucleus. To evaluate the spatial distribution of immune cells around the eggs, we analyzed 4 samples of infected Peyer’s patches. Each Peyer’s patch usually has 5–6 granulomas. We calculated the number of immune cells of each granuloma at intervals of 50 μm within the range of 0 to 400 μm from the egg. The final data for graphing is the average number of the 4 PP samples. Finally, the number of marker-positive cells per field of view (n/sight) was calculated based on the fluorescence signal distribution relative to the nucleus. The average fluorescence intensity of each positively expressed marker was calculated for quantitative analysis. Densities and counts were calculated and exported to excel.

### 2.9 Peyer’s patch dissociation and preparation

Select 5~6 Peyer’s patches from each of the HC group and the Infected group, and then perform the following steps in parallel: Cut the Peyer’s patches into as small pieces as possible and place them into a 15 ml centrifuge tube. Add 2 ml of 10 % trypsin-EDTA (25200056, Gibco) for digestion at 37°C, which should last approximately 30 minutes. During this period, continuously pipette up and down to ensure complete digestion until no obvious tissue blocks are visible. Add 2 ml of complete DMEM medium to stop the digestion. After filtering the mixture through a 100 μm cell strainer, a single-cell suspension of the PPs is prepared for subsequent single-cell sequencing.

### 2.10 10X Genomics scRNA-seq library preparation, quantification, and analysis

The cells were captured via the 10X Chromium System according to the manufacturer’s protocol. In a microfluidic chip, individual cells were combined with reaction reagents and gel beads containing cell-specific barcode sequences, which were encapsulated together in oil droplets to form gel beads-in-emulsion (GEMs). Inside the GEMs, the cells were lysed to release RNA, which bound to poly(dT) primers carrying cell barcodes and unique molecular identifiers (UMIs), under appropriate conditions, initiating complementary strand extension. Three cytosine (C) bases were added to the 3’ end of the extended strand and paired with the rGrGrG sequence of the template-switching oligo (TSO), followed by extension using the TSO as a template to complete the reverse transcription reaction. The GEMs were then disrupted, and the resulting cDNA was recovered, amplified via PCR, and used to construct the cDNA library.

PE150 sequencing data from 10X Genomics, consisting of R1 and R2 reads for each sample, were generated, and quality control analysis was performed on the R2 data via FastQC software. Cell Ranger was employed for further statistical analysis of the sequencing data, removing duplicate UMIs for each gene associated with a barcode and counting unique UMIs to quantify gene expression levels within each cell, thereby minimizing PCR artifacts. By taking into account the total count of UMIs for each barcode, barcodes with relatively higher UMIs were identified as representing high-quality cells. On the contrary, those barcodes with lower UMIs were categorized as background noise. This approach enabled the discrimination between cellular signals and non-cellular data. Seurat software was used for downstream analysis, including cell filtering, data normalization, batch effect correction, principal component analysis (PCA), t-distributed stochastic neighbor embedding (t-SNE) analysis, uniform manifold approximation and projection (UMAP) analysis, cell clustering, marker gene identification, and cell annotation.

### 2.11 Statistical analysis

Statistical analyses were performed with GraphPad Prism 9. Unpaired Student’s t-tests and two-tailed Mann–Whitney U-tests were used for two-group analysis. All general statistical analyses were performed with a confidence interval of 95%. P values ≤0.05 were considered to indicate significance (^*^P <0.05; ^**^P <0.01; ^***^P <0.001). The data are presented as the means ± SEMs, as indicated in the figures.

## 3 Results

### 3.1 *S. japonicum* eggs increase collagen in Peyer’s patches

**Figure 1:**
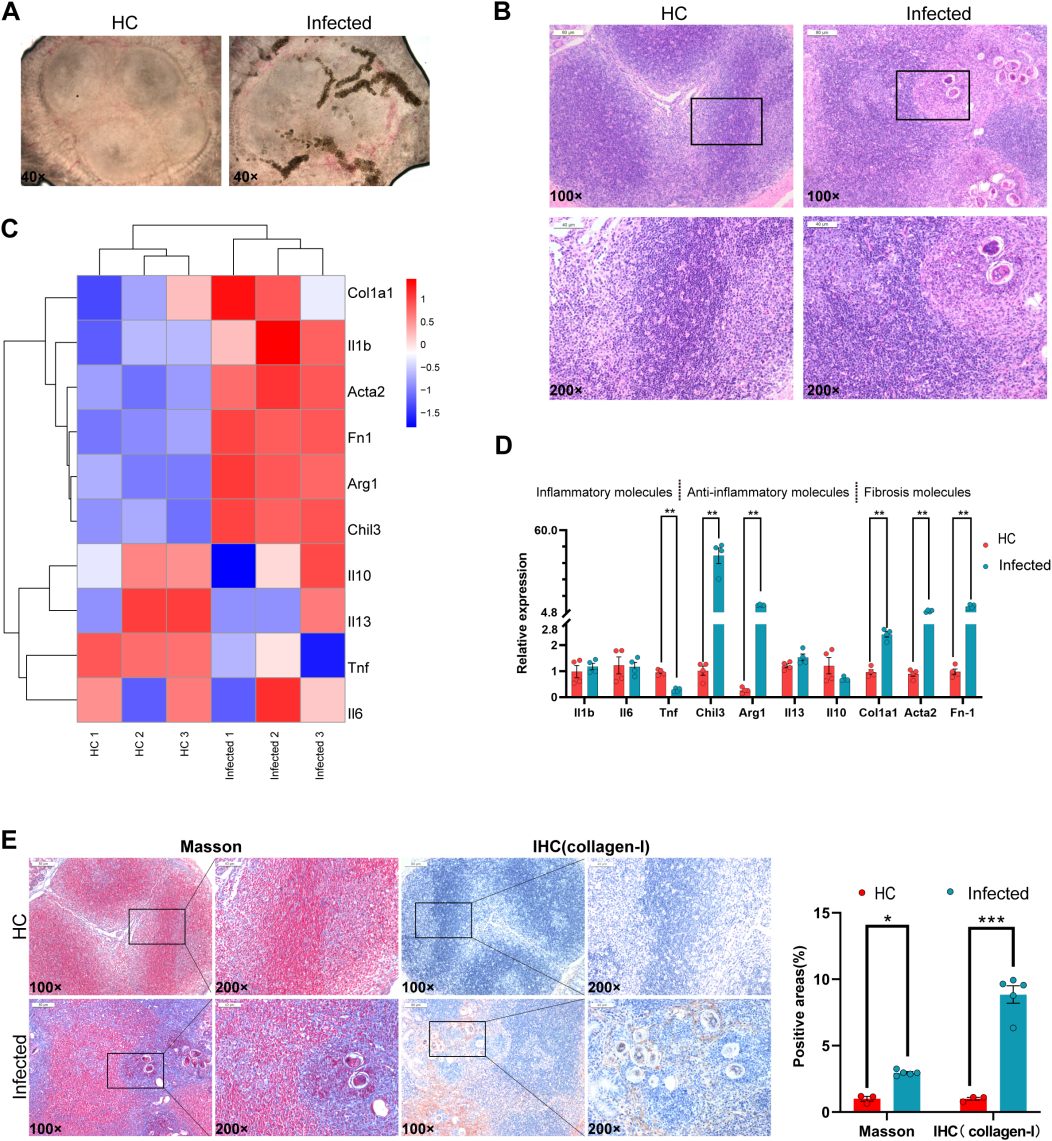
Eggs destroy the immune balance in PPs. **(A)** Egg deposition in PPs. **(B)** Hematoxylin and eosin (H&E) staining of PP sections. **(C)** RNA-seq heatmap of inflammation and fibrosis molecules. **(D)** qPCR analysis of inflammatory and fibrosis-related molecules. **(E)** Masson staining and immunohistochemistry of PPs. All data; n = 3–5, *P<0.05, **P<0.01, ***P <0.001.

Previous studies have shown that schistosome eggs can deposit within PPs along vascular systems (Turner et al., 2012); however, the structural and functional consequences of this deposition on PPs have remained unclear. To address this, we utilized a murine *S. japonicum* infection model to investigate the effects of egg accumulation on PP structure and immune function. Consistent with previous observations, we confirmed the distribution of *S. japonicum* eggs along the vascular system within PPs (**Figure 1A**), and their presence was validated by hematoxylin and eosin (H&E) staining (**Figure 1B**).

To explore the molecular changes induced by egg deposition, we performed RNA-seq and qPCR on PP tissues. The results revealed significant upregulation of key genes compared with those in the uninfected controls. Specifically, the expression of the inflammatory inhibitors *Chil3* and *Arg1* increased approximately 40-fold, indicating a modulated inflammatory response. In addition, fibrosis-related genes were markedly elevated: the expression of Col1a1 increased 2-fold, that of Acta2 increased 5-fold, and that of Fn1 increased 9-fold (**Figures 1C, D**). Histological analysis further revealed a dramatic increase in the number of collagen fibers within PPs, accompanied by a 9-fold increase in Collagen-I expression, as confirmed by immunohistochemistry and qPCR (**Figure 1E**).

These findings demonstrate that *S. japonicum* egg deposition in PPs induces a fibrotic process characterized by increased collagen deposition and significant upregulation of fibrosis-associated genes. Furthermore, egg accumulation alters the structural integrity of PPs and triggers a molecular cascade favoring fibrosis.

### 3.2 The distribution of immune cells and *S. japonicum* eggs within PPs

**Figure 2:**
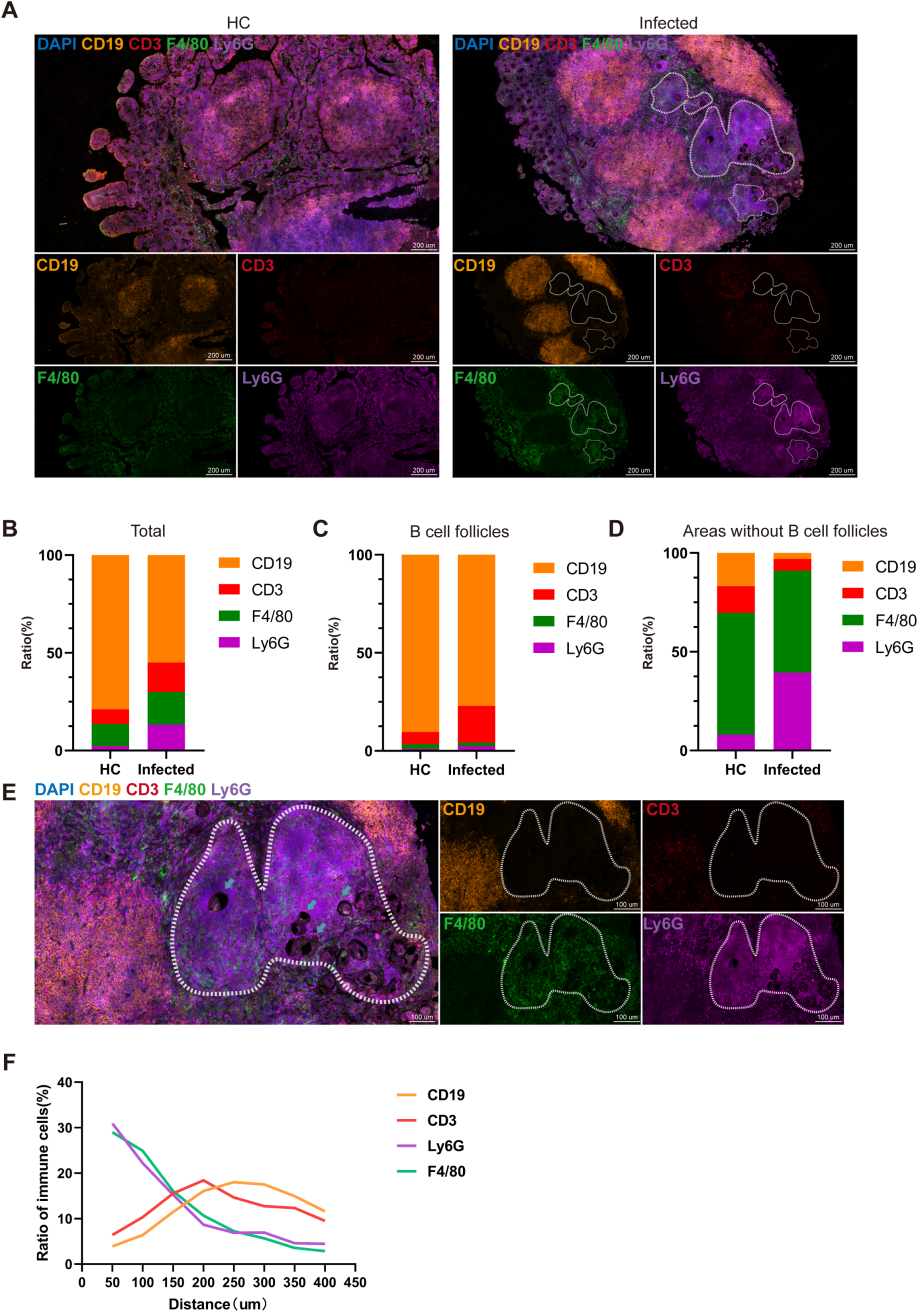
The components of granulomas in PPs. **(A)** Multiplex immunofluorescence image showing the distribution of immune cells in HC PPs and infected PPs: CD19 (yellow), CD3 (red), F4/80 (green), and Ly6G (purple). **(B)** Comparison of immune cell ratios. **(C, D)** Comparison of the immune cell ratio in B-cell follicles **(C)** and areas without B-cell follicles **(D)**. **(E)** Multiplex immunofluorescence illustrating the distribution of immune cells in granulomas. Arrows point to egg CD19 (yellow), CD3 (red), F4/80 (green), and Ly6G (purple). **(F)** Distinctive ratios of immune cells at different distances from eggs.

To investigate the spatial interaction between *S. japonicum* eggs and immune cells within PPs, we utilized mIF to visualize the following: B cells (CD19^+^), T cells (CD3^+^), macrophages (F4/80^+^), neutrophils (Ly6G^+^), and *S. japonicum* eggs. In healthy controls (HCs), PPs were predominantly composed of B cells, T cells, macrophages, and neutrophils (**Figure 2A**). Compared with HCs, infected PPs presented a significant increase in the number of macrophages, neutrophils, and T cells, with T cells and neutrophils showing the most pronounced increase (**Figures 2A, B**). Quantitative analysis further revealed a marked increase in neutrophils specifically within the non-B-cell follicular zones of infected PPs relative to the B-cell follicular zones (**Figures 2C, D**).

Many studies have explored the differences in the cellular composition and spatial distribution of *Schistosoma* egg granulomas in the liver and intestines (Schwartz and Fallon, 2018). Previous studies have indicated that neutrophils are the primary responders in granuloma formation—recruiting macrophages and initiating a mixed T helper (Th) response through the formation of neutrophil extracellular traps (NETs) (Chuah et al., 2013; Wang et al., 2022). Consistent with those findings, our results demonstrated that macrophages and neutrophils were concentrated in the inner layer of the granulomas surrounding *S. japonicum* eggs, while T cells and B cells were located on the periphery of the granulomas (**Figure 2E**). In addition, by observing the cellular distribution around the eggs using the eggs as the center and in units of 50 μm, it was found that within a distance of 0–150 μm from the eggs, the number of neutrophils and macrophages was three times greater than that of B and T cells. The number of T cells reached a maximum of 200 μm, whereas the number of B cells gradually increased above 200 μm. These results confirmed the structure of the intestinal granulomas induced by *S. japonicum* eggs and the spatial distribution of immune cells (**Figure 2F**).

### 3.3 Deposition of eggs leads to the apoptosis of immune cells in PPs

To investigate the cellular composition of PPs in response to *S. japonicum* infection, we performed scRNA-seq on PPs isolated from one healthy mouse and one infected mouse. This analysis generated a total of 15,190 single-cell transcriptomes. Details of the quality control procedures are provided in the **Supplementary Materials** (**Supplementary Figure S1**). To our knowledge, this study represents the first application of scRNA-seq to map the cellular distribution of PPs in the context of schistosome infection, building on prior work (Zheng et al., 2020; Wang et al., 2022).

**Figure 3:**
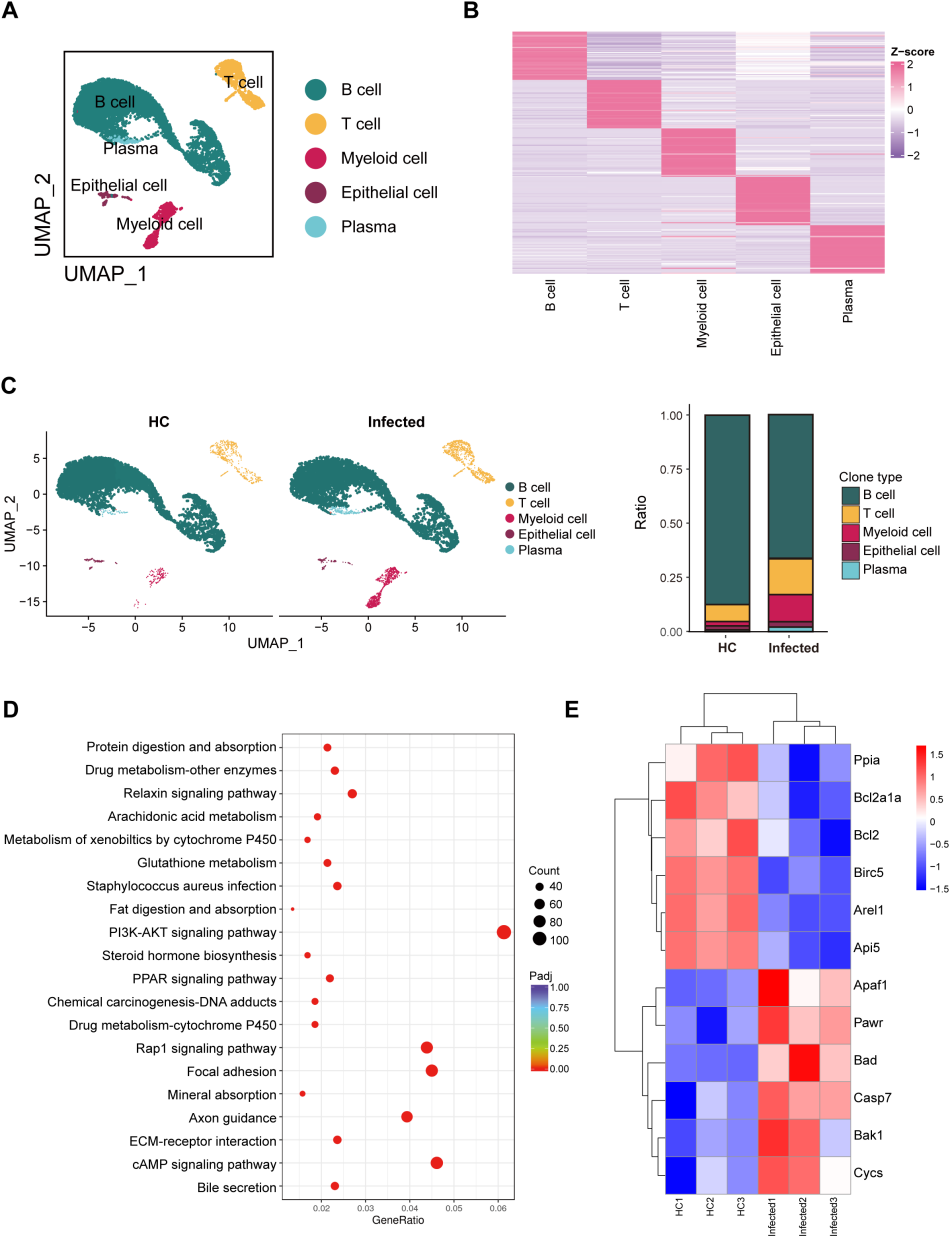
Overview of single-cell transcriptional profiling of PPs and RNA-seq of PPs revealing cell survival status. **(A)** UMAP visualization of B cells, T cells, myeloid cells, and plasma. **(B)** Heatmap visualization of the top 50 genes in five cell types. **(C)** Distribution of different cells in HCs and infected mice. **(D)** KEGG enrichment analysis was conducted on the RNA-seq data of Peyer’s patches (PPs) from healthy controls (HCs) and infected mice. The PI3K-AKT signaling pathway was found to be significantly enriched in the infected mice. **(E)** Heatmap visualization of apoptosis genes based on the RNA-seq data of PPs.

Using UMAP visualization, we analyzed the combined dataset from the HC and infected samples and identified five major cell clusters: B cells, T cells, myeloid cells, plasma cells (PCs), and epithelial cells (**Figure 3A**). These clusters were defined according to the expression of established marker genes, with the top 50 marker genes for each cluster visualized via differential expression analysis (**Figure 3B**). Notably, dendritic cells (DCs) were not detected in our dataset, likely due to technical limitations during cell isolation or sequencing.

Our analysis revealed distinct changes in cell proportions between the healthy and infected groups (**Figure 3C**). Specifically, the proportion of B cells decreased in the infected group, while the proportions of T cells and myeloid cells increased. These shifts were consistent with independent measurements obtained via Tissue FAXS, reinforcing the validity of our scRNA-seq findings. Notably, as shown in **Figure 2A**, the numbers of multiple immune cell types, including B cells, increase following infection. Thus, the decrease in the proportion of B cells is more likely attributable to the increase in other cell types, such as neutrophils.

To explore the molecular mechanisms underlying these cellular changes, we conducted the Kyoto Encyclopedia of Genes and Genomes (KEGG) pathway enrichment analysis. The results highlighted significant activation of the PI3K/AKT signaling pathway in the infected group (**Figure 3D**). This pathway is associated with the regulation of apoptosis. Further examination revealed upregulation of proapoptotic genes, including *Apaf1*, *Pawr*, *Bad*, *Casp7*, *Bak1*, and *Cycs*, alongside downregulation of antiapoptotic genes, such as *Ppia*, *Bcl2a1a*, *Bcl2*, *Birc5*, *Arel1*, and *Api5* (**Figure 3E**). These gene expression changes suggest that the deposition of *S. japonicum* eggs in PPs triggers apoptosis, particularly affecting B cells, which aligns with the observed reduction in their proportion.

### 3.4 B-cell function is suppressed, and B cells tend to undergo apoptosis

**Figure 4:**
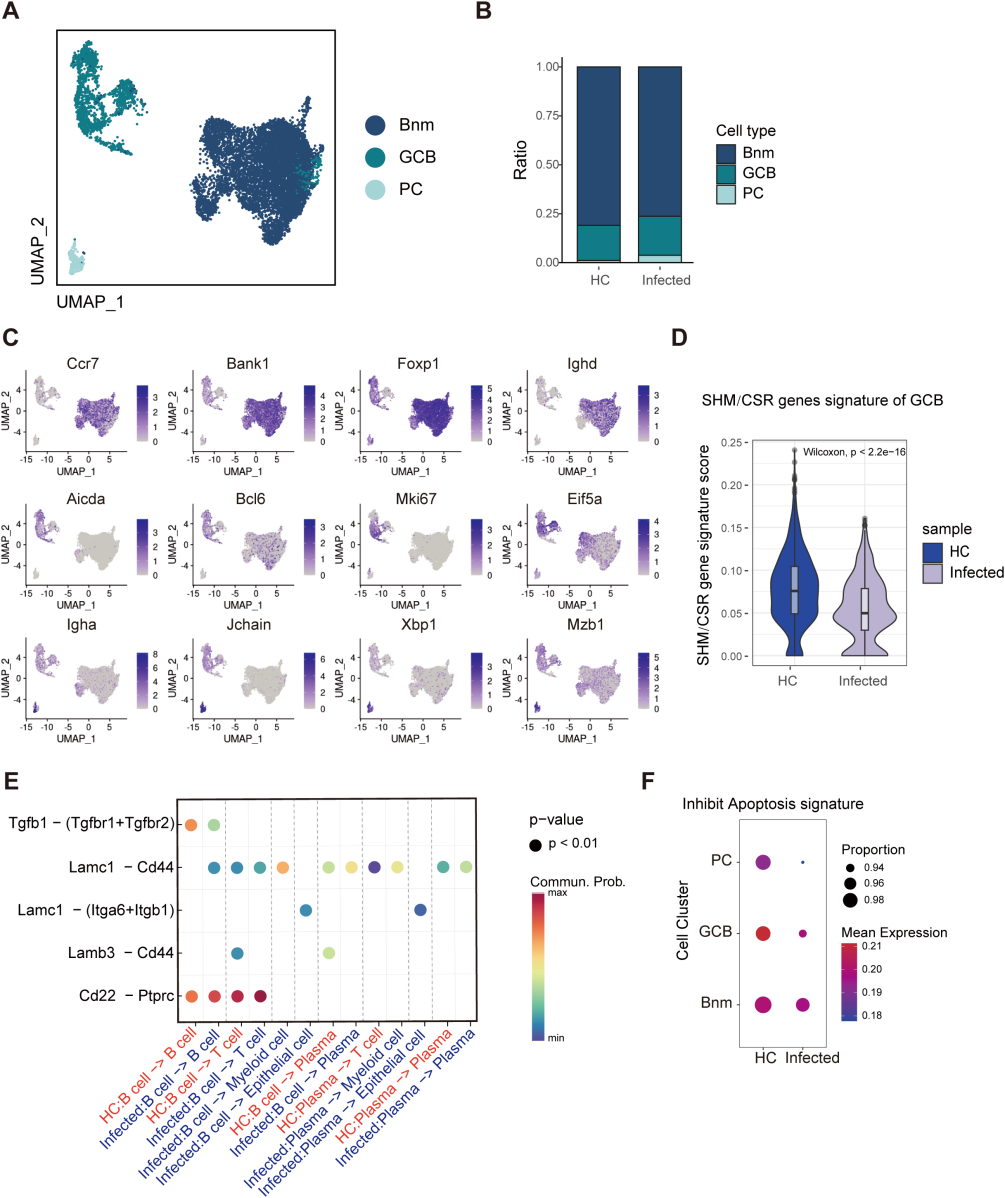
Single-cell RNA-seq analysis reveals B-cell inhibition and apoptosis. **(A)** UMAP visualization of three B-cell subclusters: naïve B cells and memory B cells (Bnm), germinal center B cells (GCB), and plasma cells (PCs). **(B)** Distributions of Bnm, GCB, and PCs. **(C)** UMAP visualization of the expression of marker genes. **(D)** Violin plot of SHM and CSR genes in GCB. These genes were down-regulated in infected mice. **(E)** Analysis of B-cell interactions. The interaction of CD22-PTPCR was enhanced in infected mice. **(F)** Circle plot of apoptosis genes in B cells. Decreased expression of inhibitors of apoptosis genes was observed in infected mice.

B cells are essential for the formation of granulomas in *S. japonicum* infection; however, it remains unclear whether granulomas reciprocally affect B-cell function (Wang et al., 2022). To investigate this, we selected and reclustered 9913 B cells and plasma cells (PCs) via scRNA-seq based on classical gene markers. We identified three distinct subsets: naïve or memory B cells (Bnm) (*Ccr7, Bank1, Foxp1, Ighd*), germinal center-like B cells (GCB) (*Aicda, Bcl6, Mki67, Eif5a*), and plasma cells (PCs) (*Igha, Jchain, Xbp1, Mzb1*) (**Figures 4A, C**) (Glaros et al., 2021; Wang et al., 2021; Uzzan et al., 2022). Additionally, in infected mice, although the proportions of Bnm, GCB, and PCs in infected mice were not significantly different with HC (**Figure 4B**), genes associated with somatic hypermutation (SHM) and class-switch recombination (CSR) were downregulated in GCBs (**Figure 4D**), suggesting a potential reduction in antibody diversity. In addition, we observed increased expression of CD22, a known suppressor of B-cell activation (Masamba and Kappo, 2021; Mörbe et al., 2021) (**Figure 4E**), and decreased expression of inhibitors of apoptosis proteins (**Figure 4F**). These findings indicate that B cells in infected mice are prone to apoptosis and exhibit impaired function.

### 3.5 Th2 cells dominate the exhaustion of T cells

**Figure 5:**
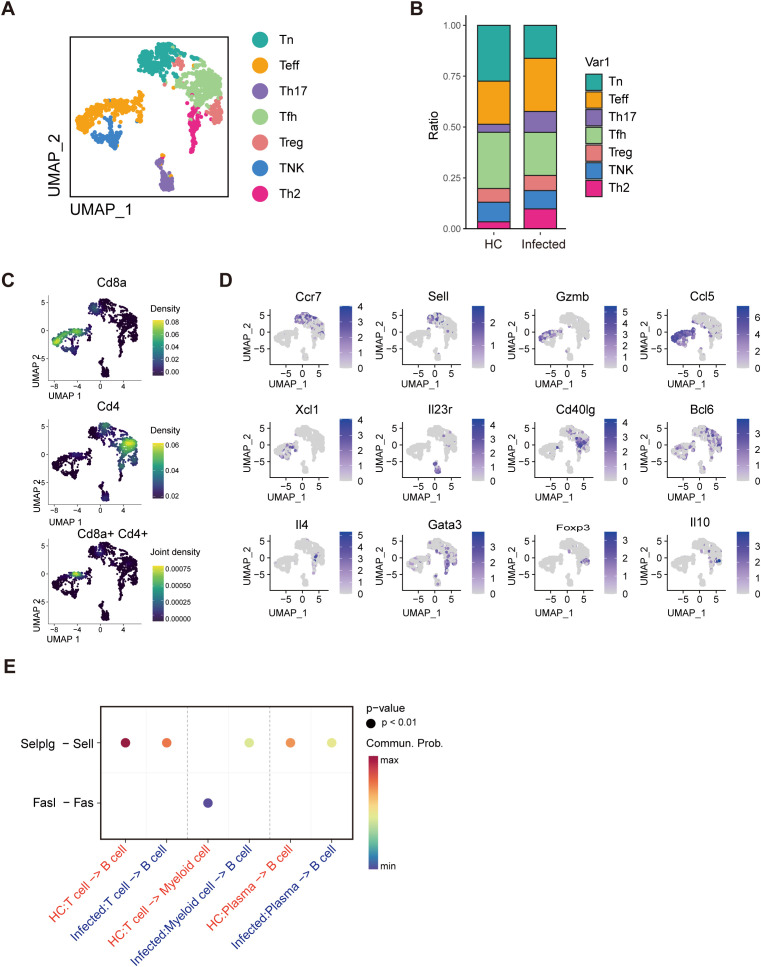
Single-cell RNA-seq analysis reveals T-cell exhaustion. **(A)** UMAP visualization of seven T-cell subclusters: naïve T cells (Tn), T helper 17 (Th17), T helper 1 (Th1), T helper 2 (Th2), follicular helper T (Tfh), effective T (Teff), and NK-like T (TNK) cells. **(B)** Distributions of Tn, Th17, Th1, Th2, Tfh, Teff, and TNK cells. **(C)** UMAP visualization of the expression of CD4^+^ and CD8^+^ marker genes. **(D)** UMAP visualization of the expression of T-cell marker genes. **(E)** Analysis of T-cell interactions. The interaction between T-cell and B-cell was attenuated.

T lymphocytes are broadly classified into CD4^+^ helper T (Th) cells and CD8^+^ cytotoxic T lymphocytes (CTLs), with Th cells playing a pivotal role in orchestrating humoral and cellular immune responses against parasitic infections (Wang et al., 2022). To evaluate the impact of *S. japonicum* egg deposition in PPs on T-cell responses, we characterized T-cell subsets via single-cell RNA sequencing (scRNA-seq). Subsets were defined according to canonical marker gene expression: naïve T cells (Tn, *Ccr7*), Th17 cells (*Il22*), Th2 cells (*Gata3*), follicular helper T cells (Tfh, *Bcl6*), regulatory T cells (Treg, *Foxp3*), effector T cells (Teff, *Gzmb*), and NK-like T cells (TNK, *Xcl1*) (**Figures 5A, D**).

Analysis of CD4^+^ T-cell populations revealed distinct subset profiles; however, some overlap in gene expression between the Tn and Teff subsets resulted in partial mixing of CD4^+^ and CD8^+^ cells (**Figure 5C**). Compared with those from healthy controls (HCs), PPs from infected mice presented a significantly greater proportion of Th2 cells, with no detectable Th1 cells (**Figure 5B**). This marked Th2 dominance suggests an immunosuppressive microenvironment in the infected PPs. Furthermore, weakened interactions between Selplg and Sell (**Figure 5E**) indicate T-cell exhaustion (Mörbe et al., 2021), reflecting compromised T-cell functionality in response to egg deposition.

### 3.6 Weak interaction between T cells and B cells

**Figure 6:**
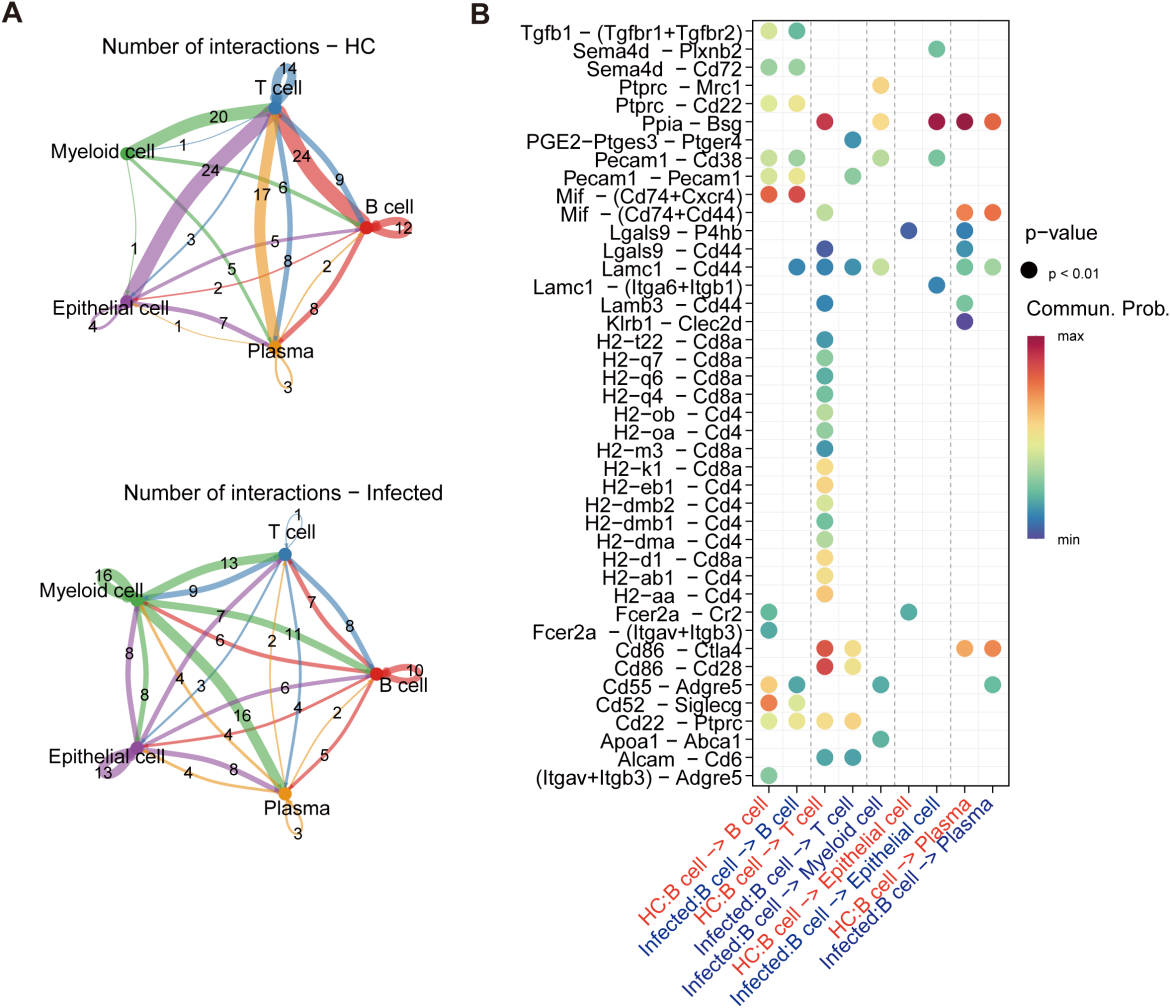
Cell interaction reveals a decline in T–B-cell interplay. **(A)** The number of interactions in the cell–cell communication network. T-cell–B-cell (B–T) interactions were the most severely affected, 24 interactions in healthy controls but only 7 in the infected mice. **(B)** Analysis of B-cell interactions. The interaction between MHC molecules on B cells and the CD4 or CD8 complexes on T cells was completely abolished.

We analyzed intercellular signaling pathways in healthy controls (HCs) and infected individuals via the CellChat algorithm to investigate the interactions among distinct immune cell types. A summary chord diagram was generated to quantify the number of ligand–receptor interactions between each pair of cell populations. Notably, in infected mice, interactions between T cells and all other cell types were significantly diminished, with the total number of interactions reduced by approximately half. Among these interactions, T-cell–B-cell (B–T) interactions were the most severely affected, exhibiting a >2-fold decrease (**Figure 6A**). Strikingly, the interaction between MHC molecules on B cells and the CD4 or CD8 complexes on T cells was completely abolished, suggesting that T cells cannot receive the initial signal required for activation (**Figure 6B**).

### 3.7 Fibrosis signaling pathway activation in myeloid cells

**Figure 7:**
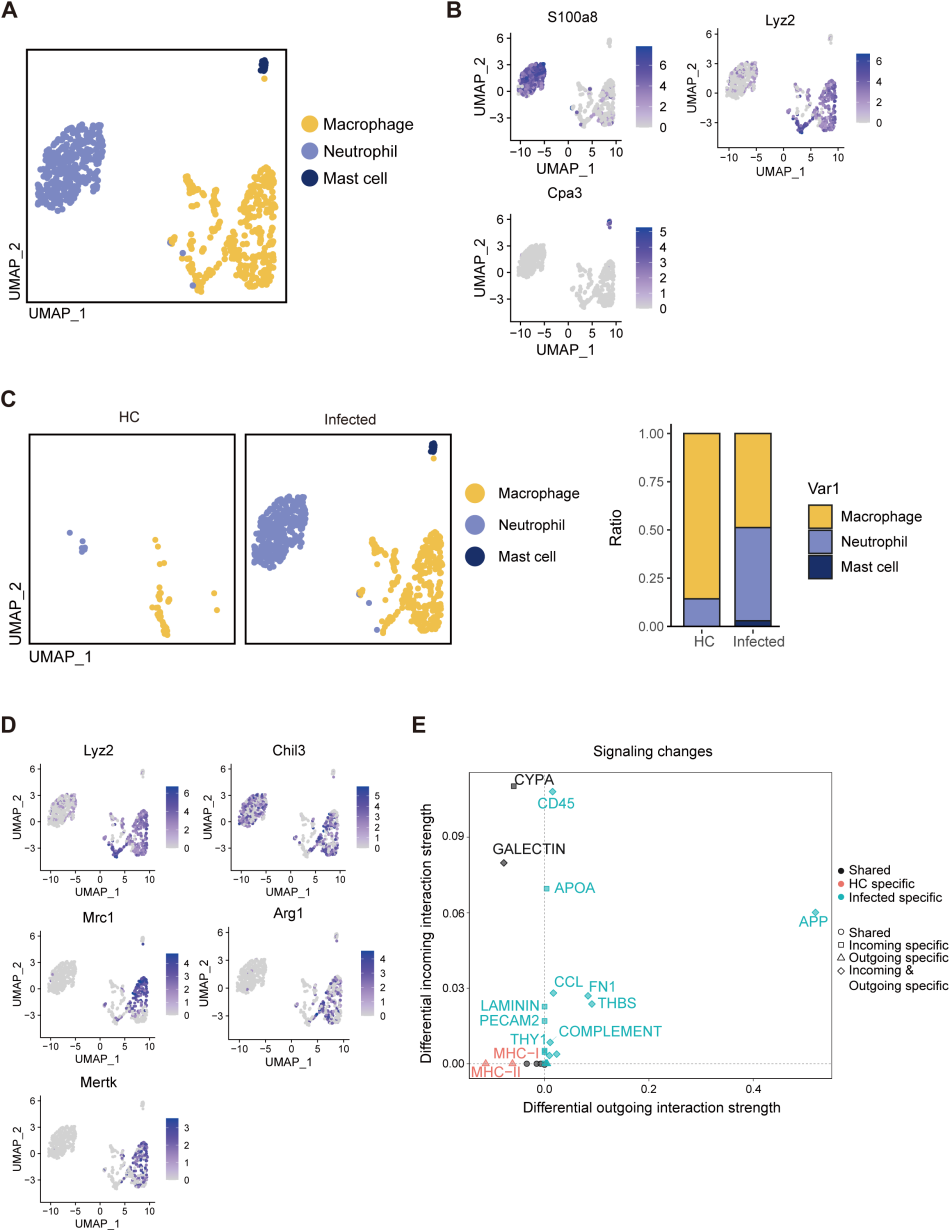
Single-cell RNA-seq analysis reveals that myeloid cells activate fibrosis signaling. **(A)** UMAP visualization of three myeloid cell subclusters: macrophages, neutrophils, and mast cells. **(B)** Expression of marker genes via UMAP visualization. **(C)** Distributions of macrophages, neutrophils, and mast cells. **(D)** UMAP visualization of the expression of M2 marker genes. **(E)** Analysis of signaling changes in myeloid cell interactions.

A total of 1672 myeloid cells, comprising macrophages, neutrophils, and mast cells, which are characterized by the expression of Lyz2, S100a8, and Cpa3, respectively, were identified as the most rapidly expanding cell types (**Figures 7A, B**). In contrast to the minimal presence of macrophages and neutrophils observed in healthy controls (HCs), both populations increased substantially following egg deposition. Notably, the number of neutrophils increased approximately twofold (**Figure 7C**), which is consistent with prior studies identifying neutrophils as the initial responders to *S. japonicum* eggs. Conversely, eosinophils have been recognized as the principal initial responders to *S. mansoni* eggs. Given the immunosuppressive state observed in PPs, we attempted to classify macrophages according to classical inflammatory phenotypes but were unable to delineate distinct subgroups of classically activated (M1) or alternatively activated (M2) macrophages. Nevertheless, M2-associated markers (*Lyz2, Chil3, Mrc1, Arg1*, and *Mertk*) were highly expressed across the macrophage cluster, which aligns with our observation of a predominant anti-inflammatory response in the infected PPs (**Figure 7D**). Furthermore, cell–cell interaction analysis revealed active signaling via the FN1 and THBS1 pathways, both of which are implicated in fibrosis. This finding potentially explains the increased collagen deposition observed in the PP region of *S. japonicum*-infected individuals (**Figure 7E**).

Fibrosis occurs in infected PPs. However, the underlying causes of this physiological condition remain unclear. To address this, we compared signaling differences between HCs and the infected group. As anticipated, fibrosis-associated signaling pathways, including THBS, FN1, and ICAM, were markedly upregulated in the infected PP region, emerging from negligible levels in HCs (**Supplementary Figure S2A**). We examined variations within distinct cell subsets to pinpoint the source of these signaling changes. Subsequent analyses revealed that FN1, THBS, and ICAM signaling were predominantly activated in myeloid cells (**Supplementary Figure S2B**), suggesting that the recruitment of myeloid cells is the primary driver of the fibrotic process in the infected PP region.

## 4 Discussion

In summary, our study demonstrated that the deposition of *S. japonicum* eggs induces an immunosuppressive state in PPs and compromises their functional integrity. To our knowledge, this is the first report of the direct effects of *Schistosoma* on lymphoid tissues function and the first application of single-cell RNA sequencing to characterize the cellular composition and function of PPs comprehensively. Furthermore, we also validating the cellular composition of intestinal granulomas caused by *S. japonicum* infection.

**Figure 8:**
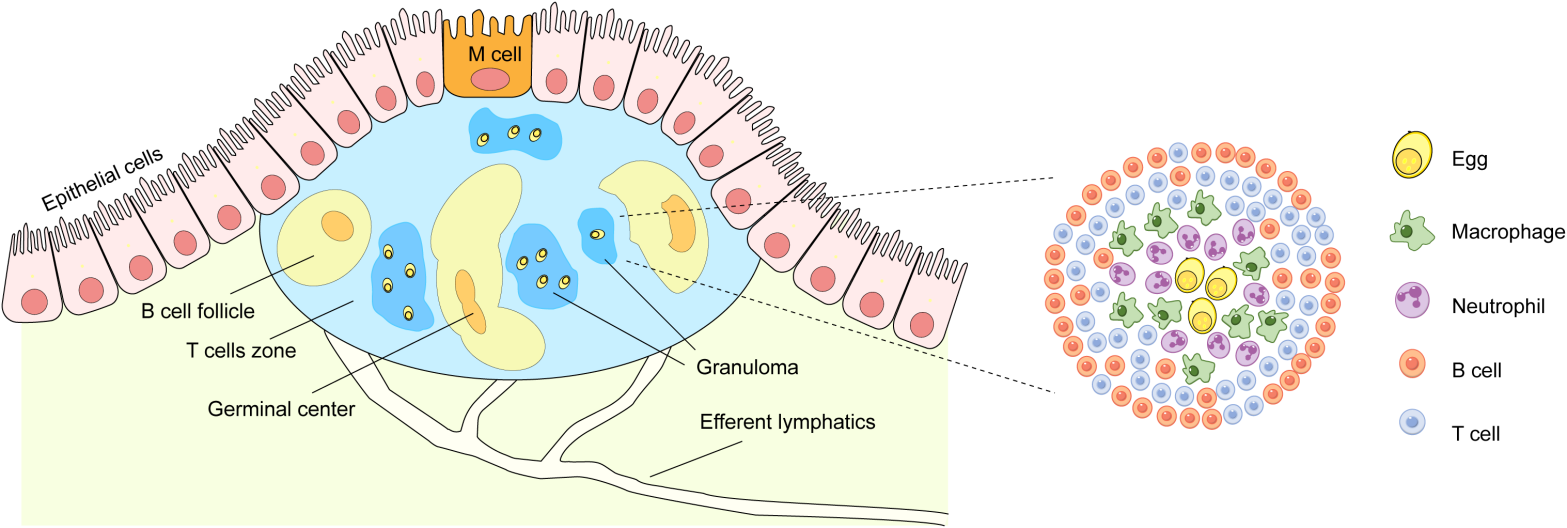
Pattern diagram for egg deposition in PPs. Worm eggs disrupt the lymphoid follicle structure in PPs and recruit neutrophils, macrophages, T cells, and B cells to form granulomatous structures. Within these structures, neutrophils and macrophages are located in the inner layer, whereas B cells and T cells occupy the outer layer.

Under physiological conditions, PPs predominantly comprise specialized epithelial cells (M cells), B cells, T cells, dendritic cells (DCs), and macrophages resembling conventional DCs in terms of key marker expression, as well as fibroblastic reticular cells (FRCs) that collectively form the PP immune niche, with no detectable neutrophils (Masamba and Kappo, 2021; Mörbe et al., 2021; Wang et al., 2022). These observations align with our findings. A significant finding (**Figure 8**) is that after the eggs fall into the PPs, they form a granuloma structure in the non-B cell follicular area and disrupt the regular structure of the B cell follicular area, resulting in a chaotic arrangement of the immune regions. The center of the egg granuloma consists of neutrophils and macrophages, and their numbers are almost similar. The number of T cells gradually increases towards the periphery, while B cells are mainly located in the outermost layer.

Our results highlighted significant activation of the PI3K/AKT signaling pathway in PPs of the infected group. The PI3K/AKT/mTOR pathway, a critical regulator of cellular metabolism, modulates apoptosis by inhibiting proapoptotic factors such as Bcl-2 antagonist of cell death (Bad), Bcl-2-like protein 11 (BIM), caspase-9, and forehead box protein O1 (FoxO1) (Wu et al., 2022). Evidence shows that upregulation of the PI3K/AKT signaling pathway can activate the autophagy regulator mTOR, inhibit autophagy, and exacerbate endothelial cell apoptosis, thereby promoting pulmonary fibrosis (Liu et al., 2019). Oxidative stress and endoplasmic reticulum stress induce alveolar epithelial apoptosis by activating the PI3K/AKT pathway, leading to the fibrosis development (Lee et al., 2020). Thus, we inferred that the activation of the PI3K/AKT pathway in the context of *S. japonicum* egg deposition in PPs plays a role in promoting apoptosis and potentially exacerbates the pathological processes related to the granuloma formation and subsequent tissue damage, similar to its role in some apoptotic and fibrotic situations described in previous studies.

Chronic granulomatous disease has been shown to diminish the production of immature B cells by the bone marrow, resulting in B-cell lineage exhaustion (Clark and Giltiay, 2018). B cells are pivotal immune constituents of PPs. The downregulation of genes associated with hypermutation (SHM) and class-switch recombination (CSR) in schistosome infected germinal center-like B cells would affect the diversity of naïve B cells, leading to decreased antibody diversity and impaired GC function.

CD22 is defined as an inhibitory receptor for B-cell function and can physically bind to the B-cell receptor (BCR) (Nitschke et al., 1997). Once SHP-1 is recruited to CD22, it dephosphorylates key signaling molecules in the BCR signaling pathway, such as Igα/Igβ and Syk, inhibiting BCR signal transduction and restricting B-cell activation (Collins et al., 2004). In contrast, BCR cross-linking on CD22^–/–^ B cells induces elevated responses (Otipoby et al., 1996; Sato et al., 1996). The upregulation of CD22 in schistosome-infected B cells suggested the existence of a regulatory mechanism aimed at dampening B-cell activation. This upregulation might prevent over-activation of B cells that could potentially lead to excessive inflammation.

PTPRC, also known as CD45, is a receptor-type protein tyrosine phosphatase widely expressed on the surface of leukocytes and is crucial for antigen receptor signaling in T and B cells (Sato et al., 1996). CD45 can activate Src family kinases through dephosphorylation, thereby initiating the activation signaling cascade of immune cells (Ashwell and D’Oro, 1999). After CD45 binds to CD22, it can regulate the conformation and function of CD22 and enhance its ability to recruit SHP-1. Compared to Heathy Control (HC) group, the CD22-PTPRC interaction between B cells in Infected group were significantly enhanced, further inhibiting the activation of B cells (**Figures 4E**, **6B**).

The immune response and immunopathology of schistosomiasis stem from CD4^+^ T-cell reactivity to egg antigens. In the acute phase of infection, parasite antigens elicit a robust Th1 response marked by elevated levels of proinflammatory cytokines, including TNF-α, IL-1, and IL-6 (Prados et al., 2021). By contrast, Th2 cells are anti-inflammatory and play a key role in granulomatous inflammation (Prados et al., 2021). The Th2 cytokine IL-13, which drives granulomatous inflammation and fibrosis via IL-4Rα signaling, has been shown to mitigate established fibrosis in schistosome infection when inhibited (Long et al., 2021). In *S. japonicum* infection, an increase in Th2 cells correlates with an anti-inflammatory state and a fibrotic trend in PPs. In addition, P-selectin glycoprotein ligand-1 (PSGL-1, encoded by SELPLG), identified as an immune checkpoint in tumor immunity, inhibits T-cell proliferation (Prieto-Bermejo et al., 2021), whereas Selplg-deficient T cells exhibit functional exhaustion (Dong et al., 2020). Recent evidence suggests that the SELPLG ligand SELL is associated with the abundance of activated B cells (Nitschke, 2009), indicating that SELPLG-SELL interactions may regulate T–B-cell crosstalk in an immunosuppressive milieu. Consistent with prior studies (Nitschke, 2005), we found that *S. japonicum* infection disrupts lymphoid follicle architecture, with further analysis revealing markedly weakened B-cell–T-cell interactions (**Figure 5E**).

During infection, *S. japonicum* eggs enter PPs via high endothelial venules (HEVs) (Turner et al., 2012). Inflammatory mediators activate endothelial cells through pattern recognition receptors (PRRs), triggering a neutrophil recruitment cascade that draws neutrophils to the vicinity of eggs (Hiatt et al., 1979). Neutrophils, in turn, recruit monocytes to inflamed tissue, resulting in both proinflammatory and anti-inflammatory phenotypes that induce M1 and M2 macrophage polarization to shape the immune response (Kaplan et al., 1998; Johnston et al., 2019). Our data revealed elevated expression of M2 marker genes (Arg1, Chil3, and Mrc1), indicating that macrophages in egg-laden PPs are predominantly M2 polarized. Although PP macrophages are typically negative for F4/80 under normal conditions (Mörbe et al., 2021), the infected group presented high F4/80 expression, suggesting recruitment from the blood circulation. Notably, some F4/80-positive macrophages were detected in our control group, likely due to the inclusion of intestinal tissue in these samples. Although eosinophils in the small intestine also express F4/80, granulomas induced by *S. japonicum* eggs are predominantly composed of neutrophils rather than eosinophils. Thus, we postulate that F4/80-positive cells are mainly macrophages (Hsü et al., 1972; Von Lichtenberg et al., 1973; Burke et al., 2010; Seki et al., 2012; Chuah et al., 2013).

Fibrosis is usually associated with factors such as impaired tissue regeneration and local tissue hypoxia (Wynn and Vannella, 2016). Impaired vascular regeneration caused by egg deposition may be the main reason for the development of PP fibrosis. Fibrosis is closely related to the function of myeloid cells. Previous studies have shown that M2 macrophages can secrete pro - fibrotic cytokines such as transforming growth factor - β (TGF - β) and platelet - derived growth factor (PDGF), which promote the activation of fibroblasts, leading to the production of large amounts of collagen, FN1, etc., and causing excessive deposition of extracellular matrix (ECM) (Buechler et al., 2021). Meanwhile, fibroblasts recruit macrophages to the fibrotic or injured sites through colony - stimulating factor 1 (CSF1) and CCL2, promoting fibrosis progress (Meziani et al., 2018). Furthermore, studies have demonstrated that macrophages promote fibroblast activation via FN1 signaling (Viramontes et al., 2022) and interact with epithelial cells through the APP–CD74 axis to drive fibrosis (Ji et al., 2008). In addition, neutrophils inhibit their own apoptosis by binding to ICAM-1 on the membrane of T84 human epithelial cells and increase epithelial cell proliferation through Akt and β-catenin signaling (Sumagin et al., 2016). Neutrophils can also secrete neutrophil elastase, which can promote the proliferation of fibroblasts and the differentiation of myofibroblasts, playing an important role in promoting tissue repair and fibrosis (Gregory et al., 2015). Additionally, eosinophils in *S. mansoni* infection have also been proven to migrate to the vicinity of the eggs via CXCR3 (Li et al., 2004) and promote fibrosis through Galectin-receptor interaction (Huang et al., 2024). Hence, egg-recruited myeloid cells may significantly contribute to collagen accumulation and fibrotic tendencies in PPs, although their potential interactions with FRCs and role in PP fibrosis warrant further investigation.

This study employed multi-omics approaches to investigate schistosome egg-induced alterations in the structure and function of PPs. However, it has several limitations. First, while the selected 42-day post-infection time point represents a well-established acute phase in schistosome research, the exclusion of chronic infection stages limits our understanding of the temporal dynamics of PP remodeling. Second, the study did not perform comparative analyses between intestinal PP responses and hepatic granuloma formation, which would have provided valuable insights into organ-specific immunopathological mechanisms. Most importantly, while our omics data revealed potential mechanisms underlying PP functional suppression (e.g., CD22 upregulation and germinal center disorganization), these findings require further experimental validation.

In conclusion, the layered cellular architecture of intestinal granulomas in PPs suggests a unique immune microenvironment of egg-driven immunosuppression and fibrotic remodeling, and the identification of fibrotic pathways in myeloid cells provides a potential therapeutic target to alleviate fibrosis in patients with *S. japonicum* infection.

## Data Availability

All data is available in the main text or the supplementary materials. The raw and processed high-throughput sequencing data of this study have been deposited in Genome Sequence Archive (GSA) database under the following accession numbers: CRA021171 (https://bigd.big.ac.cn/gsa/browse/CRA021171).

## Supplementary material

Supplementary Figure 1 Filtering for quality control. **(A)** Results before and after filtering in HCs. **(B)** Results before and after filtering in the infected state.

Supplementary Figure 2 Fibrosis signaling occurs only in myeloid cells. **(A)** Signaling pathway flow of the HC and infected groups. **(B)** Differential signaling pathways identified via UMAP visualization.
