# Supplementary material for: Egg-driven immunosuppression and granuloma zonation in Peyer’s patches of mice with Schistosoma japonicum infection
Source: Front Cell Infect Microbiol. 2025 Apr 29;15:1587166. doi: 10.3389/fcimb.2025.1587166 (PMC12070193; doi:10.3389/fcimb.2025.1587166)
Supplement: Supplementary Figure 1 — Filtering for quality control. (A) Results before and after filtering in HCs. (B) Results before and after filtering in the infected state. [file DataSheet1.pdf]

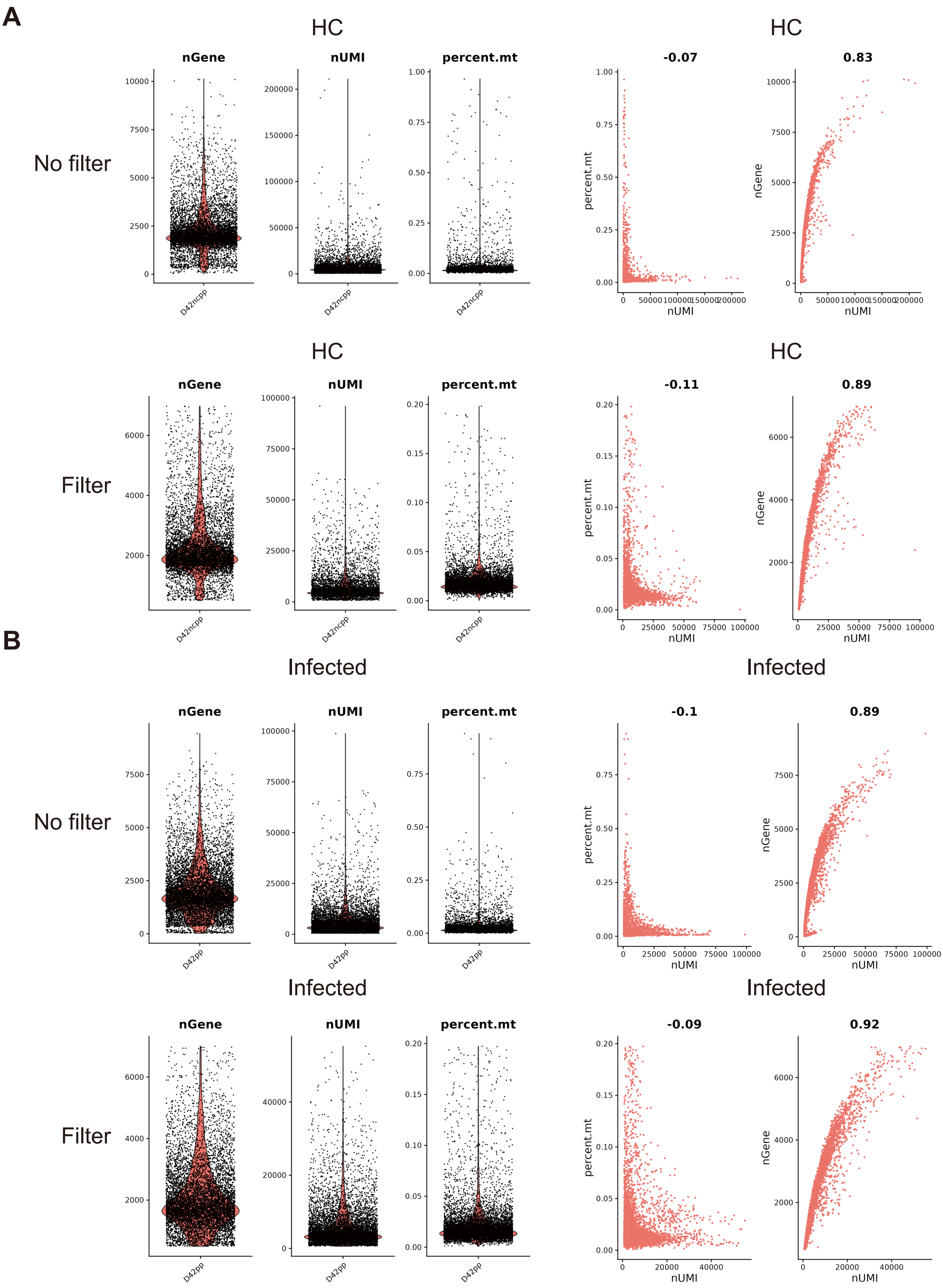

Supplementary Figure S1

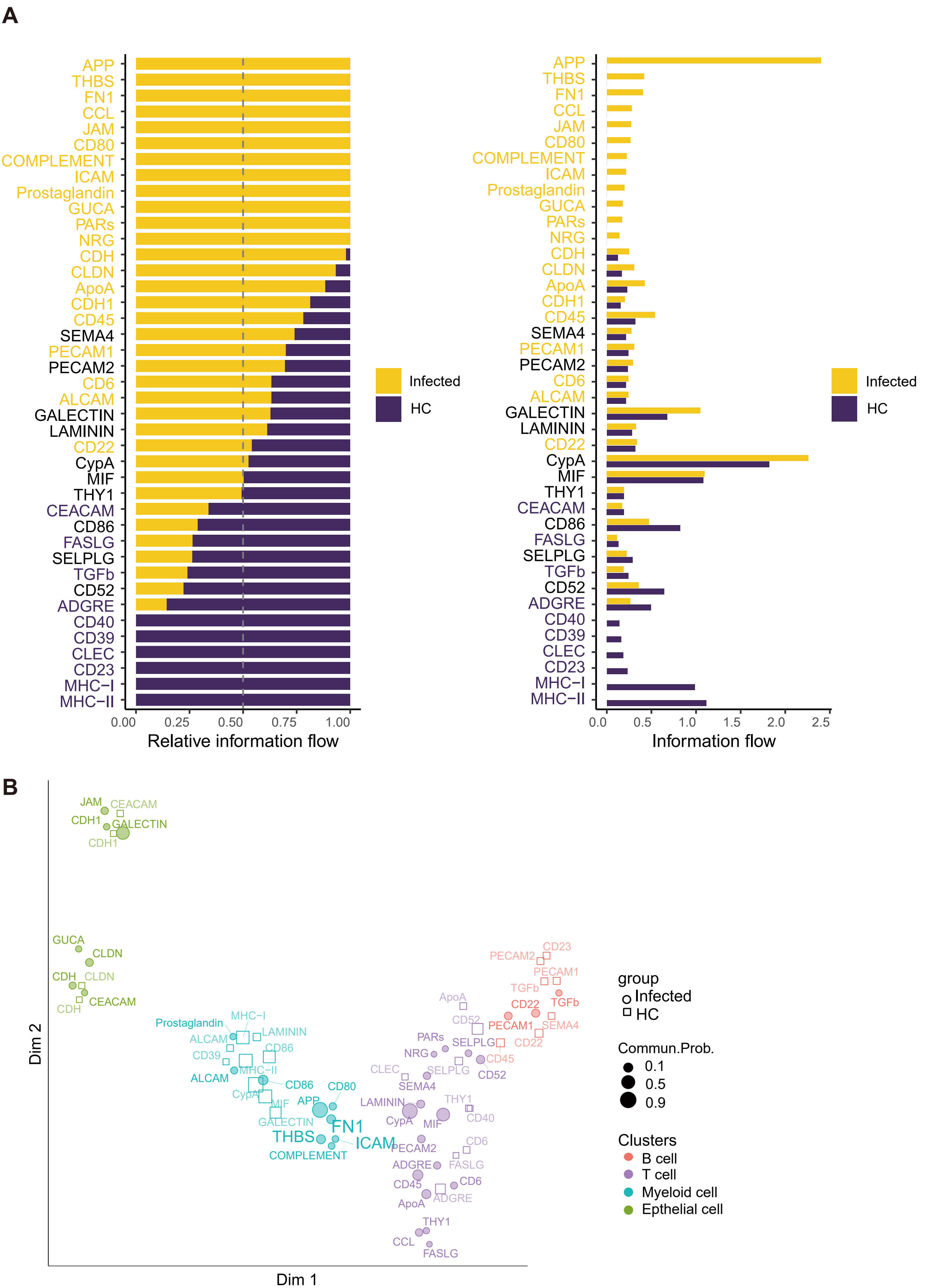

Supplementary Figure S2

Table S1 Primer Sequence

Genes	Primer	Sequence(5'-3')
Tnfa	Forward primer	CGTCAGCCGATTTGCTATCT
	Reverse primer	CGGACTCCGCAAAGTCTAAG
IL-1b	Forward primer	GCTCTCCACCTCAATGGACAGA
	Reverse primer	TGCTTGGGATCCACACTCTCC
Arg-1	Forward primer	CTCCAAGCCAAAGTCCTTAGAG
	Reverse primer	AGGAGCTGTCATTAGGGACATC
Ym1	Forward primer	CAAGTTGAAGGCTCAGTGGCTC
	Reverse primer	CAAATCATTGTGTAAAGCTCCTCT
IL6	Forward primer	TAGTCCTTCCTACCCCAATTTCC
	Reverse primer	TTGGTCCTTAGCCACTCCTTC
Il13	Forward primer	TGCAACGGCAGCATGGTATG
	Reverse primer	GAGGCTGGAGACCGTAGTGG
IL-10	Forward primer	ACTGCACCCACTTCCCAGTC
	Reverse primer	GCTTGGCAACCCAAGTAACCC
Collagen-I	Forward primer	TCCTGCGCCTAATGTCCACCGA
	Reverse primer	AAGCGACTGTTGCCTTCGCCTC
Acta2	Forward primer	CACAGCCCTGGTGTGCGACAAT
	Reverse primer	TTGCTCTGGGCTTCATCCCCCA
Fn-1	Forward primer	ATGTGGACCCCTCCTGATAGT
	Reverse primer	GCCCAGTGATTTCAGCAAAGG
